# Palmitoyl-L-carnitine induces tau phosphorylation and mitochondrial dysfunction in neuronal cells

**DOI:** 10.1371/journal.pone.0313507

**Published:** 2024-11-13

**Authors:** Gwangho Yoon, Min Kyoung Kam, Young Ho Koh, Chulman Jo

**Affiliations:** Division of Brain Disease Research, Department for Chronic Disease Convergence Research, Korea National Institute of Health, Cheongju-si, Chungcheongbuk-do, Republic of Korea; Nathan S Kline Institute, UNITED STATES OF AMERICA

## Abstract

Alzheimer’s disease (AD) is characterized by cognitive decline and memory loss, involving mechanisms such as tau hyperphosphorylation and mitochondrial dysfunction. Increasing evidence suggests that age-related alterations in metabolite levels are crucial for the pathogenesis of AD. Here, we analyzed serum metabolites from mice of various ages (2, 4, 14, and 21 months old) using mass spectrometry. We identified palmitoyl-L-carnitine as a key metabolite with significantly increased levels in aged mice. In vitro experiments with SH-SY5Y neuronal cells demonstrated that palmitoyl-L-carnitine treatment enhanced tau phosphorylation, increased mitochondrial fission, and elevated intracellular calcium levels. Furthermore, the increased levels of tau phosphorylation were significantly reduced by the inhibition of GSK-3β, CDK5, and calpain, indicating that tau kinases activated by calcium overload are directly involved in the increase of tau phosphorylation. Considering that mitochondrial fission is related to mitochondrial dysfunction, we propose that the elevated level of serum palmitoyl-L-carnitine during aging contributes to AD pathology through these pathways. These findings highlight the significant role of lipid metabolism in neurodegeneration and offer potential therapeutic targets for age-related diseases, including AD.

## Introduction

Aging is the primary risk factor for Alzheimer’s disease (AD), a progressive neurodegenerative disorder characterized by cognitive decline and memory loss [1]. The intricate interplay between aging and AD involves various biological mechanisms, including the accumulation of amyloid-beta plaques due to the abnormal processing of amyloid precursor proteins, formation of neurofibrillary tangles from hyperphosphorylated tau proteins, mitochondrial dysfunction leading to oxidative stress, and chronic inflammation propelled by activated macrophages and microglia [2, 3]. Aging also contributes to synaptic dysfunction, impaired protein homeostasis, calcium dysregulation, and increased permeability of the blood-brain barrier [4–7]. Moreover, age-related genetic and epigenetic changes, coupled with lifestyle factors and comorbidities such as hypertension and diabetes, further increase the susceptibility to AD [8–10]. However, the complexity of interactions between these processes makes them challenging to fully understand, highlighting the need to explore the underlying causes that contribute to these complex interdependencies.

Accumulating evidence suggests that age-related alterations in metabolite levels play a pivotal role in the complex pathogenesis of AD [11]. Elevated ceramide levels, which are associated with cognitive decline and brain atrophy, are notable in aging individuals and patients with AD [12]. Phosphatidylcholine levels, which are crucial for maintaining cell membrane integrity, are decreased in AD and correlate with neuronal dysfunction and tau pathology [13]. Age-related reductions in branched-chain amino acids affect protein synthesis and energy metabolism, which exacerbate AD pathology [14]. Increased serum homocysteine levels, a common occurrence during aging, contribute to oxidative stress, inflammation, and tau hyperphosphorylation, which aggravate cognitive decline [15, 16]. Alterations in short-chain fatty acids due to shifts in the gut microbiota influence systemic inflammation and brain function, highlighting the role of the gut-brain axis in aging and neurodegeneration [17, 18]. Impaired glucose metabolism, evidenced by diminished glycolytic intermediates, indicates mitochondrial dysfunction and decreased neuronal energy availability, both of which are prominent features of AD [19]. Furthermore, markers of oxidative stress, such as 4-hydroxynonenal, increase with age, reflecting the oxidative damage that exacerbates AD progression [20, 21]. Elevated pro-inflammatory cytokines like IL-6, TNF-α, and IL-1β are also linked with aging, fostering neuroinflammation and tau pathology in AD [22]. Therefore, investigating the broader spectrum of aging-related metabolite alterations holds promise for enhancing our understanding of the etiology of AD and potentially revealing biomarkers for early diagnosis and targets for therapeutic interventions.

In the present study, we aimed to investigate how age-related changes in metabolite levels contribute to AD pathology, with a specific focus on the role of palmitoyl-L-carnitine. We examined the serum metabolite levels in mice across various age groups using mass spectrometry and identified palmitoyl-L-carnitine as a key metabolite with significantly increased levels in aged mice. Palmitoyl-L-carnitine was chosen due to its known involvement in mitochondrial metabolism of palmitic acid, which is implicated in AD pathology [23]. We found, for the first time, that palmitoyl-L-carnitine could induce tau phosphorylation and mitochondrial dysfunction in neuronal cells. This discovery not only strengthens the association between AD and metabolites but also underscores the potential of metabolites as promising therapeutic targets.

## Materials and methods

### Animals

Wild-type (C57BL/6J) mice obtained from the Jackson Laboratory [24], were used in this study. These mice were housed at the Laboratory Animal Research Center of the Korea National Institute of Health under controlled conditions: a 16-h light/8-h dark cycle, 23°C temperature, and humidity levels of 60 ± 10%. The mice had free access to food and water and were used in experiments at 4, 14, and 21 months of age. All procedures, including preliminary experiments, followed protocols approved by the Institutional Animal Care and Use Committee (IACUC Approval No. KDCA-IACUC-24-023) and adhered to the Korea National Institute of Health guidelines for laboratory animal care and use. No human subjects were involved in this study, and no additional ethical approvals were required beyond those for animal care and use.

### Metabolomic analysis

Metabolomic analysis was performed using AbsoluteIDQ p180 (Biocrates) following the protocol described in a previous study [25] with slight modifications. Blood serum from aged mice was prepared following the manufacturerʹs guideline. First, 5 μL of blood serum was transferred to the top 96-well plate and dried under a nitrogen stream. Next, 50 μL of a 5% phenylisothiocyanate (PITC) solution was added to derivatize the amino acids and biogenic amines. After incubation, the filter spots were dried again before extracting the metabolites using 300 μL of 5 mM ammonium acetate in methanol from the lower 96-well plate. Samples were further diluted with MS running solvent A and prepared for analysis. Amino acids and biogenic amines were quantified using liquid chromatography coupled with tandem mass spectrometry (LC-MS/MS), while acylcarnitines, lipids and hexoses were analyzed by flow injection analysis mass spectrometry (FIA-MS/MS) on an ABI 4000 Q-Trap mass spectrometer (Applied Biosystems). Metabolite identification was confirmed by comparing the mass-to-charge ratio (m/z) and retention time with reference standards. The entire assay workflow, including sample registration, automated calculation of metabolite concentrations, and data export for further analysis, was managed with Biocrates MetIQ software. Quantification was performed using internal standards and calibration curves to ensure accuracy.

To compare serum metabolites across different ages in mice, we compared the metabolites at 4, 14, and 21 months of age against those at 2 months of age, calculating the fold changes relative to the 2-month-old group. Fold change was calculated by dividing the metabolite concentration in each of the older age groups by the concentration in the 2-month-old group. Multiple comparisons were corrected using the Ordinary one-way ANOVA with Tukey’s multiple comparison tests.

### Bovine serum albumin conjugation

Palmitoyl-L-carnitine (Sigma) was initially dissolved in absolute ethanol (Thermo Fisher Scientific) to make a 5 mM solution. The solution was heated to 40°C and kept at that temperature for at least 2 h with vortexing. Next, the palmitoyl-L-carnitine solution and negative control (absolute ethanol only) were filtered through a 0.45 μm syringe filter (Millipore). The filtered solution was mixed with a 10% pre-warmed bovine serum albumin (BSA) solution at a 1:100 ratio. Finally, the palmitoyl-L-carnitine stock solution (50 μM) was diluted to the desired concentrations for in vitro analyses.

### Drug treatment

Human SH-SY5Y cells were treated with 5 μM BSA-conjugated palmitoyl-L-carnitine (BSA-PC) for 24h, with BSA alone as the control [26]. To assess the effects of kinase inhibitors, cells were treated simultaneously with 5 μM GSK-3β inhibitor SB216763 (Tocris), 5 μM CDK5 inhibitor Roscovitine (Tocris), or 5 μM calpain inhibitor PD150606 (Tocris), each combined with 5 μM BSA-PC for 24h. All treatments maintained a DMSO solvent ratio of 1:1000. For molecular and calcium staining analyses, cells were treated with BSA alone or BSA-PC for 24 h. To investigate mitochondrial dynamics, cells were transfected with the GFP-Mito plasmid previously used in the study [27] for 24 h, followed by an additional 24 h treatment with BSA alone or BSA-PC.

### Cell lines and cultures

Human SH-SY5Y neuroblastoma cells were obtained from the American Type Culture Collection. Cells were cultured in DMEM/F12 supplemented with 10% fetal bovine serum and 100 U/ml penicillin-streptomycin. Cultures were maintained at 37°C in a 5% CO_2_ atmosphere, with medium changes every two days. Sub-culturing was done with pre-warmed 1X PBS and 0.25% trypsin (both Thermo Scientific). For molecular analyses and imaging, 6- and 12-well plates were seeded at 2 × 10^4^ cells/cm^2^. Experiments began 24 h after seeding.

### Mitochondrial dynamics imaging

For mitochondrial visualization, human SH-SY5Y cells were transfected with the GFP-Mito plasmid using Lipofectamine 3000 (Thermo Fisher Scientific) according to the manufacturer’s protocol. Mitochondrial dynamics were assessed 48 h after transfection. GFP-Mito-transfected cells were fixed in 2% paraformaldehyde (Sigma-Aldrich) for 15 min. After three washes with 1×PBS, the cells were mounted with the ProLong Gold Antifade Reagent (Thermo Fisher Scientific). Fluorescence images were captured with an EVOS M5000 microscope (Invitrogen).

### Calcium staining

Cytosolic calcium in SH-SY5Y cells was measured using Fluo-4 AM solution (Invitrogen) following the manufacturer’s protocol. A 3 mM Fluo-4 AM/DMSO stock solution was diluted to a 3 μM working solution in the culture medium. The cells were incubated with the Fluo-4 AM working solution for 20 min at 37°C in a CO_2_ incubator. The cells were then washed with indicator-free medium to remove non-specifically bound dyes. Cells were incubated in indicator-free medium for an additional 30 min to ensure complete de-esterification of the intracellular AM esters. Calcium imaging was done with an EVOS M5000 microscope (Invitrogen).

### Western blot analyses

Cells were lysed in ice-cold RIPA buffer (GenDEPOT) for 10 min on ice. Protein concentrations were measure with a BCA assay kit (Thermo Fisher Scientific) according to the manufacturer’s instructions. Protein samples (15–25 μg) were separated on 8–12% pre-made sodium dodecyl sulfate-polyacrylamide gels (Thermo Scientific) and transferred onto polyvinylidene difluoride (Merck Millipore) membranes using absolute methanol (Thermo Fisher Scientific). Membranes were blocked in a solution of 5% BSA (GenDEPOT) and skim milk (Cell Signaling Technology) for one hour at room temperature to enhance the detection of phosphorylated and native protein forms. After blocking, membranes were incubated overnight at 4°C with primary antibodies (1:1,000 dilution). The primary antibodies included pTau (T181; CST #12885), pTau (S262; Abcam #ab131354), PHF-1 (Peter Davies Lab.), Tau5 (Peter Davies Lab.), pDRP1 (S616; CST #4494), DRP1 (CST #8570), pMFF (S146; CST #49281), MFF (CST #14739), OPA1 (CST #80471), Mitofusin-1 (CST #14739), Mitofusin-2 (CST #11925), pGSK-3β (S9; CST #5558), GSK-3β (CST #12456), CDK5 (Santa Cruz #sc-173), p35 (Santa Cruz #sc-820), p62 (CST #8025), LC3 (CST #3868), and Actin (Millipore #MAB1501). After incubation with the primary antibodies, the membranes were probed with the corresponding horseradish peroxidase-conjugated secondary antibodies (1:10,000 dilution; BioLegend) for one hour at room temperature. Protein bands were visualized with an ECL solution (Thermo Fisher Scientific) and imaged with a ChemiDoc Imaging System (BIO-RAD). Protein expression levels was quantified using ImageJ software (V1.53c, NIH), and normalized against beta-actin and respective native proteins.

### Statistical analyses

Data are presented as mean ± standard error of the mean (SEM) unless stated otherwise. Samples were randomly assigned to control or experimental groups, and the investigators were blinded to the experimental conditions. None of the samples were excluded from the analysis. Normal distribution of the data was confirmed with the Shapiro-Wilk test. Statistical comparisons between control and experimental samples were performed using unpaired two-tailed t-tests with Welch’s correction for unequal variances or ordinary two-way analyses of variance (ANOVA) with Tukey’s multiple comparison test, as appropriate. Statistical significance was defined as p < 0.05.

## Results

### Age-related alterations in serum metabolites

To identify age-associated lipid metabolites, we performed metabolomic analysis on serum samples from mice aged 2, 4, 14, and 21 months. We profiled metabolites that act as fatty acid carriers, including acylcarnitines (categorized by carbon chain length), phosphatidylcholines (PC) with diacyl (aa) or acyl-alkyl (ae) structures, and sphingomyelins (SM), in both hydroxylated and non-hydroxylated forms.

**Fig 1:**
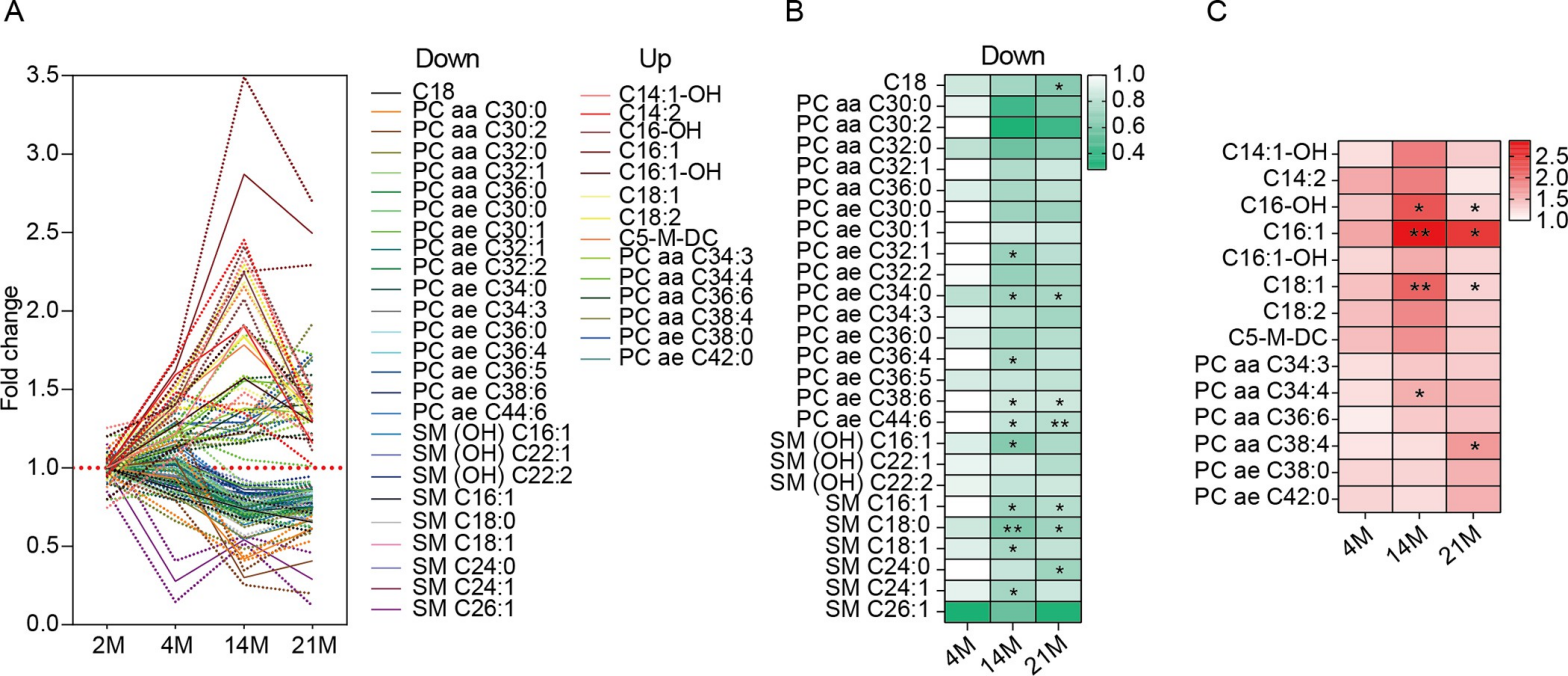
Serum metabolomic response to aging. **(A)** Metabolites (phosphatidylcholines, sphingomyelins, acylcarnitines) showing differential patterns in the serum of mice aged 2 (n = 2), 4 (n = 3), 14 (n = 2), and 21 months (n = 3). Results are shown as mean (solid line) ± standard error of the mean (SEM; dotted line). **(B)** Metabolites that decreased with aging. Statistical significance is indicated in the heatmap with asterisks. **(C)** Metabolites that increased with aging. Statistical significance is indicated in the heatmap with asterisks. In **(A)** to **(C)**, PC and SM represent phosphatidylcholine and sphingomyelin, respectively. The terms aa, ae, and -OH represent diacyl, acyl-alkyl, and hydroxylated forms, respectively. In **(B)** and **(C)**, data were analyzed with an ordinary two-way ANOVA with Tukey’s multiple comparison test; *p < 0.05, **p < 0.01.

We identified notable metabolites that increased or decreased with age (Fig 1A). Most derivatives of PC and SM decreased with age, whereas most acylcarnitines and some PCs increased. Intriguingly, PC ae C34:0, C38:6, C44:6, and SM C16:1 and C18:0 significantly decreased with age (Fig 1B). Conversely, acylcarnitines like C16-OH, C16:1, and C18:1, increased significantly with age (Fig 1C). These results suggest significant age-related changes in serum acylcarnitine, phosphatidylcholine, and sphingomyelin levels.

### Palmitoyl-L-carnitine enhances tau phosphorylation

Among the altered metabolites, acylcarnitine C16:1, which showed the most significant increase with age, was identified as palmitoyl-L-carnitine. Palmitoyl-L-carnitine is a long-chain acylcarnitine, specifically an ester of palmitic acid and L-carnitine [28]. Given the extensive literature demonstrating that palmitic acid induces post-translational modifications of tau in neurodegenerative diseases, including AD [23], we hypothesized that age-related increases in serum palmitoyl-L-carnitine influence tau phosphorylation in the brain.

**Fig 2:**
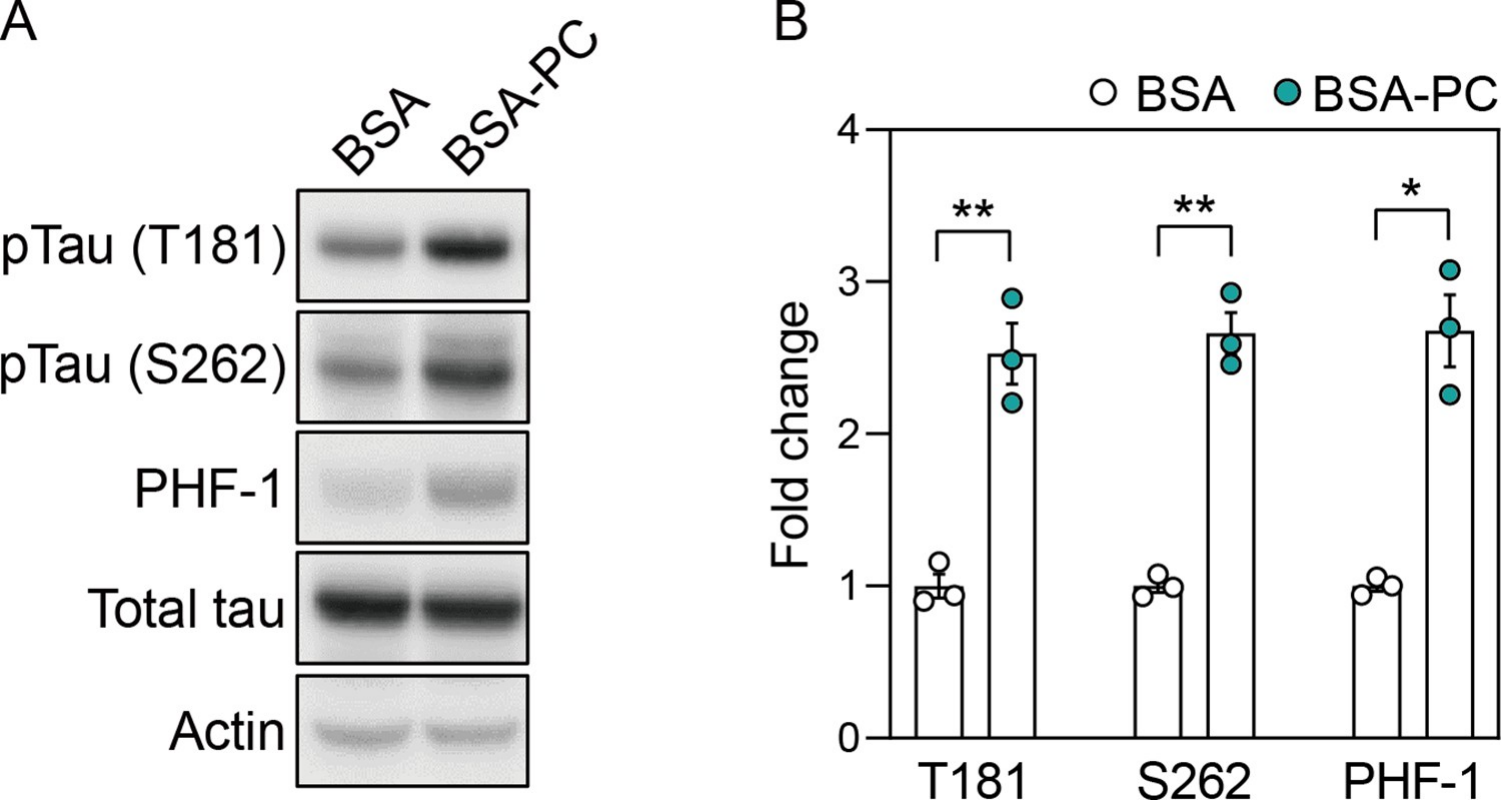
Palmitoyl-L-carnitine increases tau phosphorylation. **(A)** shows changes in protein levels of pTau (T181), pTau (S262), PHF-1, and total tau after treatment with palmitoyl-L-carnitine in SH-SY5Y cells. Full blots are provided in S1 File. **(B)** Histogram illustrating protein levels of pTau (T181), pTau (S262), and PHF-1 in cells treated with palmitoyl-L-carnitine, shown as mean ± standard error of the mean (SEM; n = 3). Tau phosphorylation levels were normalized to the total tau protein. In **(A)** and **(B)**, pTau (T181), pTau (S262), and PHF-1 represent tau phosphorylated at threonine 181, serine 262, and serine 396/404, respectively. BSA and BSA-PC represent bovine serum albumin and BSA-conjugated palmitoyl-L-carnitine, respectively. Statistical significance was determined using an unpaired two-tailed t-test with Welch’s correction; *p < 0.05, **p < 0.01.

To test this hypothesis, we conjugated palmitoyl-L-carnitine with BSA (BSA-PA) to efficiently deliver it to SH-SY5Y neuronal cells. Notably, BSA-PA significantly increased tau phosphorylation at residues T181, S262, and S396/S404 (PHF-1) compared to cells treated with BSA alone, without significant changes in total tau levels (Fig 2). This result suggests that palmitoyl-L-carnitine increases tau phosphorylation, a pathological marker of AD neurons.

### Palmitoyl-L-carnitine induces mitochondrial fission

Palmitoyl-L-carnitine is an intermediate metabolite that transports palmitic acid to the mitochondria via the carnitine shuttle for energy production [29]. Moreover, given evidence that disruptions in fatty acid metabolism or β-oxidation, such as increased serum acylcarnitines like palmitoyl-L-carnitine, affect mitochondrial dynamics and function [30, 31], we investigated whether palmitoyl-L-carnitine regulates mitochondrial fission or fusion.

**Fig 3:**
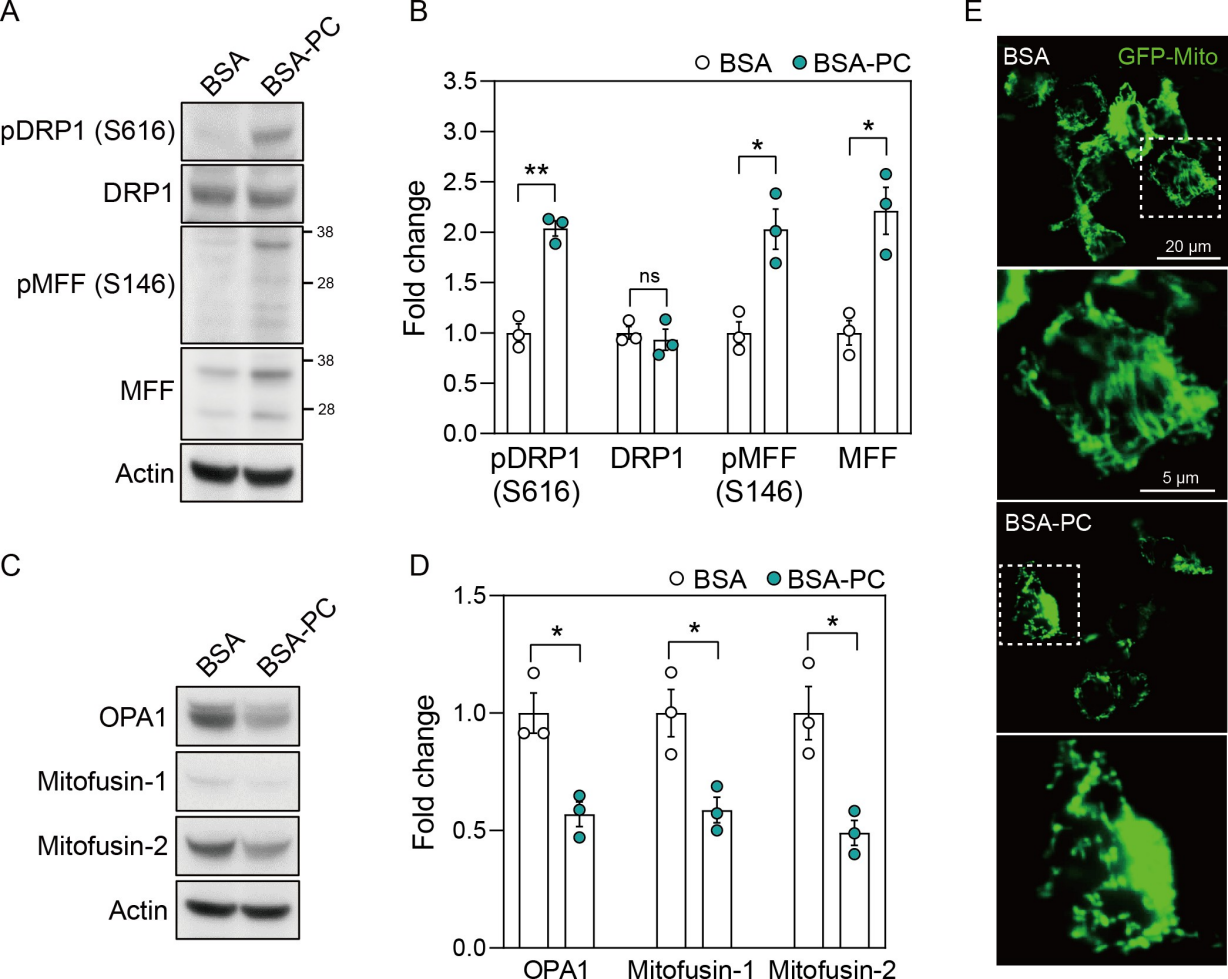
Palmitoyl-L-carnitine leads to mitochondrial fission. **(A)** shows changes in protein levels of pDRP1 (S616), DRP1, pMFF (S146), and MFF after palmitoyl-L-carnitine treatment in SH-SY5Y cells. pDRP1 (S616) and pMFF (S146) reflect DRP1 phosphorylated at serine 616 and MFF phosphorylated at serine 146, respectively. Full blots are provided in S1 File. **(B)** Histogram illustrating changes in protein levels of pDRP1 (S616), DRP1, pMFF (S146), and MFF after palmitoyl-L-carnitine treatment, shown as mean ± standard error of the mean (SEM; n = 3). **(C)** shows changes in protein levels of OPA1, Mitofusin-1, and Mitofusin-2 after palmitoyl-L-carnitine treatment in SH-SY5Y cells. Full blots are provided in S1 File. **(D)** Histogram illustrating changes in protein levels of OPA1, Mitofusin-1, and Mitofusin-2 after palmitoyl-L-carnitine treatment, shown as mean ± standard error of the mean (SEM; n = 3). **(E)** shows changes in mitochondrial dynamics after palmitoyl-L-carnitine treatment in GFP-Mito transfected SH-SY5Y cells. The images represent findings from at least three independent experiments (n = 3), with more than four fields of view analyzed per replicate. The rectangular box with a white dotted line indicates a magnified field of view. In **(A)** to **(E)**, BSA and BSA-PC represent bovine serum albumin and BSA-conjugated palmitoyl-L-carnitine, respectively. Statistical significance was determined using an unpaired two-tailed t-test with Welch’s correction; *p < 0.05, **p < 0.01.

In SH-SY5Y cells treated with BSA-PC, phosphorylation of the mitochondrial fission marker dynamin-related protein 1 (DRP1) and mitochondrial fission factor (MFF) fragments increased compared to cells treated with BSA alone (Fig 3A and 3B). The level of native MFF fragments was also higher in BSA-PC-treated cells than in BSA-treated cells, while native DRP1 levels remained unchanged. Conversely, BSA-PC markedly reduced the levels of the mitochondrial fusion markers OPA1, Mitofusin-1, and Mitofusin-2 compared to BSA-treated cells (Fig 3C and 3D).

To further validate the altered mitochondrial dynamics induced by palmitoyl-L-carnitine, SH-SY5Y cells were transfected with a GFP-Mito plasmid after treatment with BSA-PC or BSA alone. We confirmed that the elongated and interconnected mitochondrial network observed in BSA-treated cells appeared fragmented and discrete after BSA-PC treatment (Fig 3E). These results suggest that palmitoyl-L-carnitine induces mitochondrial fission, potentially leading to mitochondrial dysfunction.

### Palmitoyl-L-carnitine leads to calcium overload

**Fig 4:**
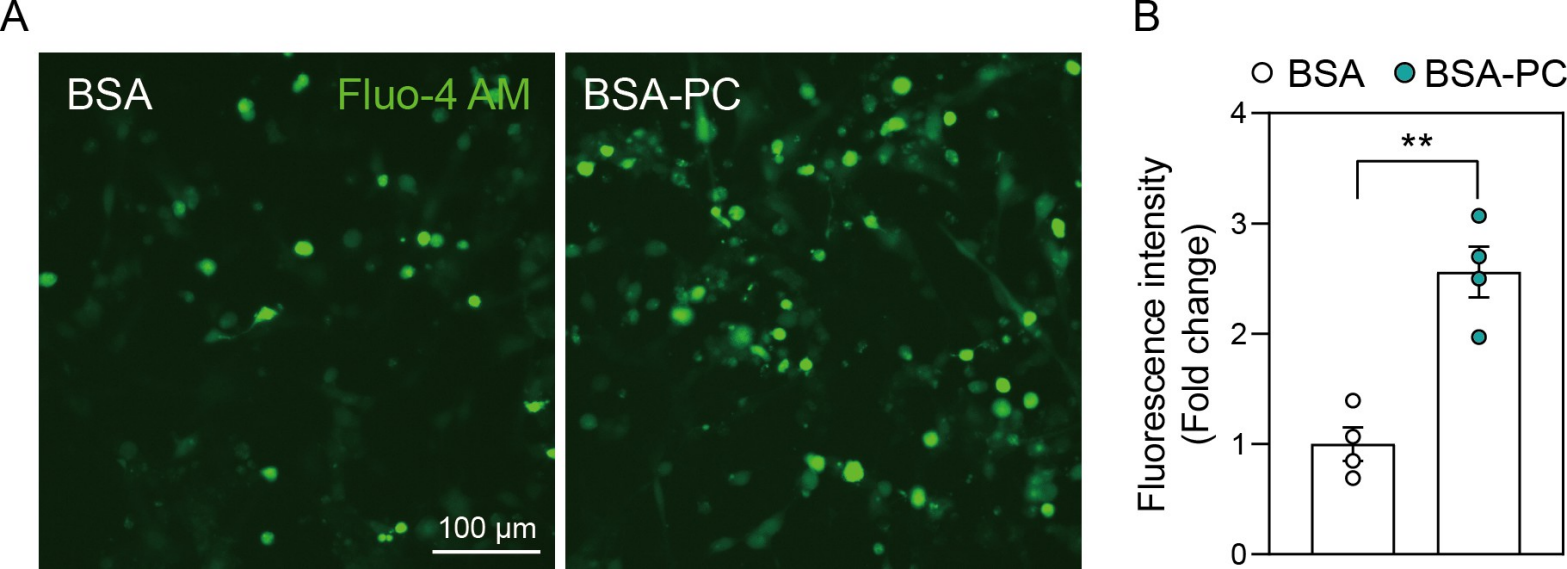
Palmitoyl-L-carnitine results in calcium overload in the cells. **(A)** shows changes in calcium overload after palmitoyl-L-carnitine treatment in SH-SY5Y cells. **(B)** shows a histogram illustrating changes in fluorescence intensity of calcium staining induced by palmitoyl-L-carnitine. Data represent three independent experiments (n = 3) and are expressed as mean ± standard error of the mean (SEM). Fluorescence intensity of calcium staining (Fluo-4 AM) was quantified using Image J software. In **(A)** and **(B)**, BSA and BSA-PC represent bovine serum albumin and BSA-conjugated palmitoyl-L-carnitine, respectively. Statistical significance was determined using an unpaired two-tailed t-test with Welch’s correction; **p < 0.01.

Some studies suggest that palmitoyl-L-carnitine can lead to calcium overload in cardiac cells [32]. Additionally, disruption of the balance between mitochondrial fission and fusion, along with impaired fatty acid metabolism, is closely linked to intracellular calcium overload in various cell types [33–35]. To test whether palmitoyl-L-carnitine induces intracellular calcium overload alongside mitochondrial fission in neurons, we treated SH-SY5Y cells with either BSA-PC or BSA alone, followed by calcium staining with Fluo-4 AM. As expected, BSA-PC significantly increased intracellular calcium overload compared to BSA alone (Fig 4). Collectively, these results suggest that palmitoyl-L-carnitine induces intracellular calcium overload and promotes mitochondrial fission.

### Palmitoyl-L-carnitine activates tau kinases

**Fig 5:**
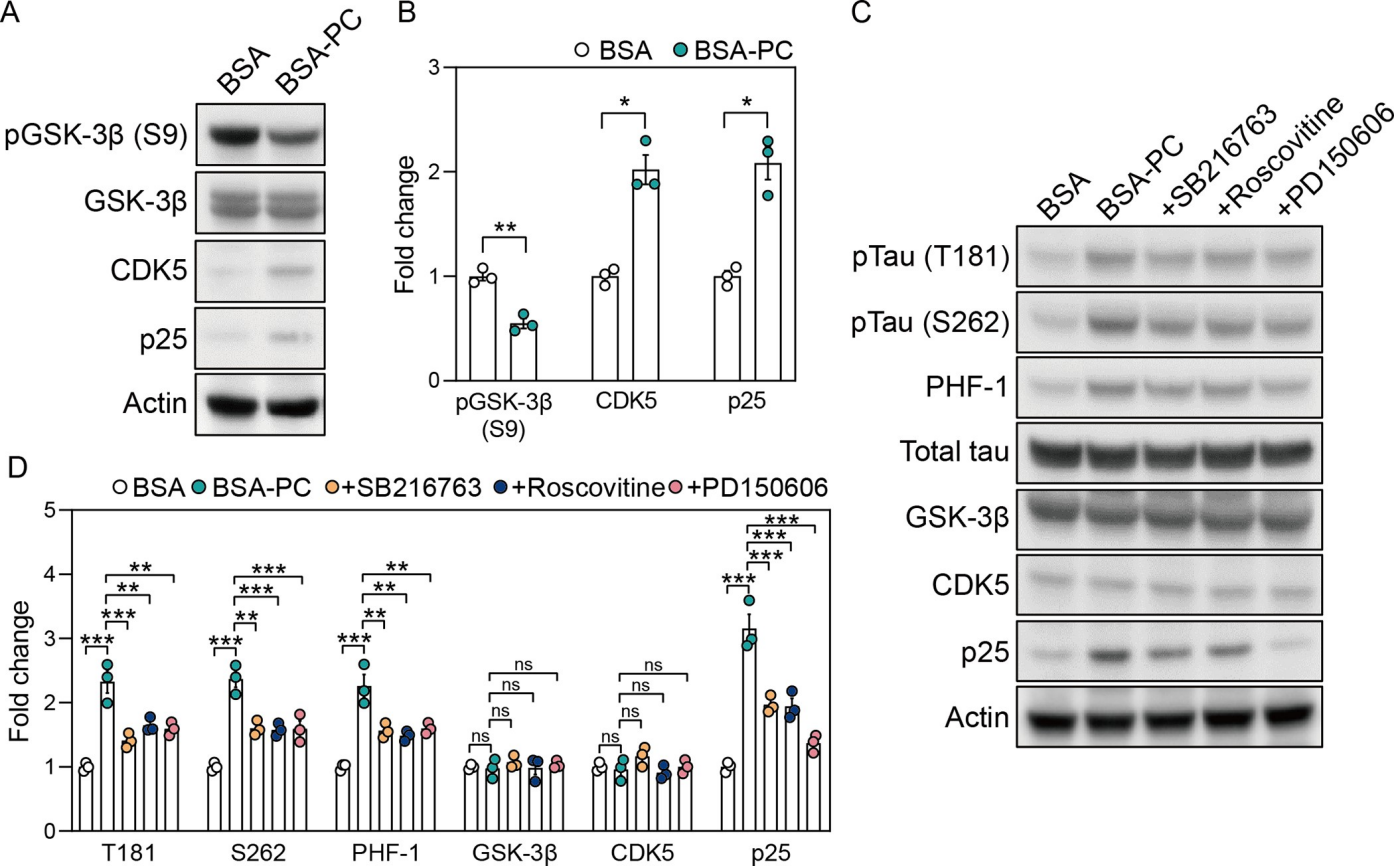
Tau kinases are involved in palmitoyl-L-carnitine-mediated tau phosphorylation. **(A)** shows changes in protein levels of pGSK-3β (S9), GSK-3β, CDK5, and p25 after treatment with palmitoyl-L-carnitine in SH-SY5Y cells. pGSK-3β (S9) indicates GSK-3β phosphorylated at serine 9. Full blots are provided in S1 File. **(B)** Histogram illustrating changes in protein levels of pGSK-3β (S9), CDK5, and p25 after treatment with palmitoyl-L-carnitine in SH-SY5Y cells, shown as mean ± standard error of the mean (SEM; n = 3). GSK-3β phosphorylation levels were normalized to the total GSK-3β protein. **(C)** shows changes in protein levels of pTau (T181), pTau (S262), PHF-1, total tau, GSK-3β, CDK5, and p25 after treatment with tau kinase inhibitors in palmitoyl-L-carnitine-treated SH-SY5Y cells. pTau (T181), pTau (S262), and PHF-1 represent tau phosphorylated at threonine 181, serine 262, and serine 396/404, respectively. SB216763, Roscovitine, and PD150606 are GSK-3β inhibitor, CDK5 inhibitor, and calpain inhibitor, respectively. Full blots are provided in S1 File. **(D)** Histogram illustrating changes in protein levels of pTau (T181), pTau (S262), PHF-1, total tau, GSK-3β, CDK5, and p25 after treatment with tau kinase inhibitors in palmitoyl-L-carnitine-treated SH-SY5Y cells, shown as mean ± standard error of the mean (SEM; n = 3). Tau phosphorylation levels were normalized to the total tau protein. In **(A)** and **(D)**, BSA and BSA-PC represent bovine serum albumin and BSA-conjugated palmitoyl-L-carnitine, respectively. Statistical significance was determined using an unpaired two-tailed t-test with Welch’s correction and an ordinary two-way ANOVA with Tukey’s multiple comparison test; ns: not significant, *p < 0.05, **p < 0.01, ***p < 0.001.

Substantial evidence indicates that calcium influx is linked to key kinases involved in tau phosphorylation. Calcium overload activates the calpain pathway, which converts p35, a CDK5 activator, into p25, thereby activating GSK-3β [36, 37]. In addition, calcium influx activates GSK-3β by dephosphorylating it at serine 9 through the calcineurin pathway [38]. Based on these findings, we investigated whether palmitoyl-L-carnitine-induced tau phosphorylation depends on the activities of GSK-3β, CDK5, and calpain. As expected, BSA-PC reduced the phosphorylation of GSK-3β at S9 in SH-SY5Y cells compared to those treated with BSA alone, without altering native GSK-3β protein levels (Fig 5A and 5B). Additionally, CDK5 and p25 levels were higher in BSA-PC-treated cells than in those treated with BSA alone. These observations suggest that the increase in tau phosphorylation induced by palmitoyl-L-carnitine is mediated by a network of tau kinase pathways associated with calcium overload.

We further demonstrated that tau phosphorylation induced by palmitoyl-L-carnitine could be mitigated by tau kinase inhibitors. SH-SY5Y cells were co-treated with BSA-PC and kinase inhibitors, including GSK-3β inhibitor SB216763, CDK5 inhibitor Roscovitine, and calpain inhibitor PD150606. These kinase inhibitors inhibited the BSA-PC-induced increase in tau phosphorylation at T181, S262, and PHF-1 sites without altering GSK-3β and CDK5 expression levels (Fig 5C and 5D). Additionally, these inhibitors reduced the BSA-PC-induced increased in p25 levels, with the calpain inhibitor having the most significant effect. Together, these findings indicate that tau phosphorylation induced by palmitoyl-L-carnitine is mediated by GSK-3β, CDK5, and calpain, which are activated by calcium overload.

## Discussion

**Fig 6:**
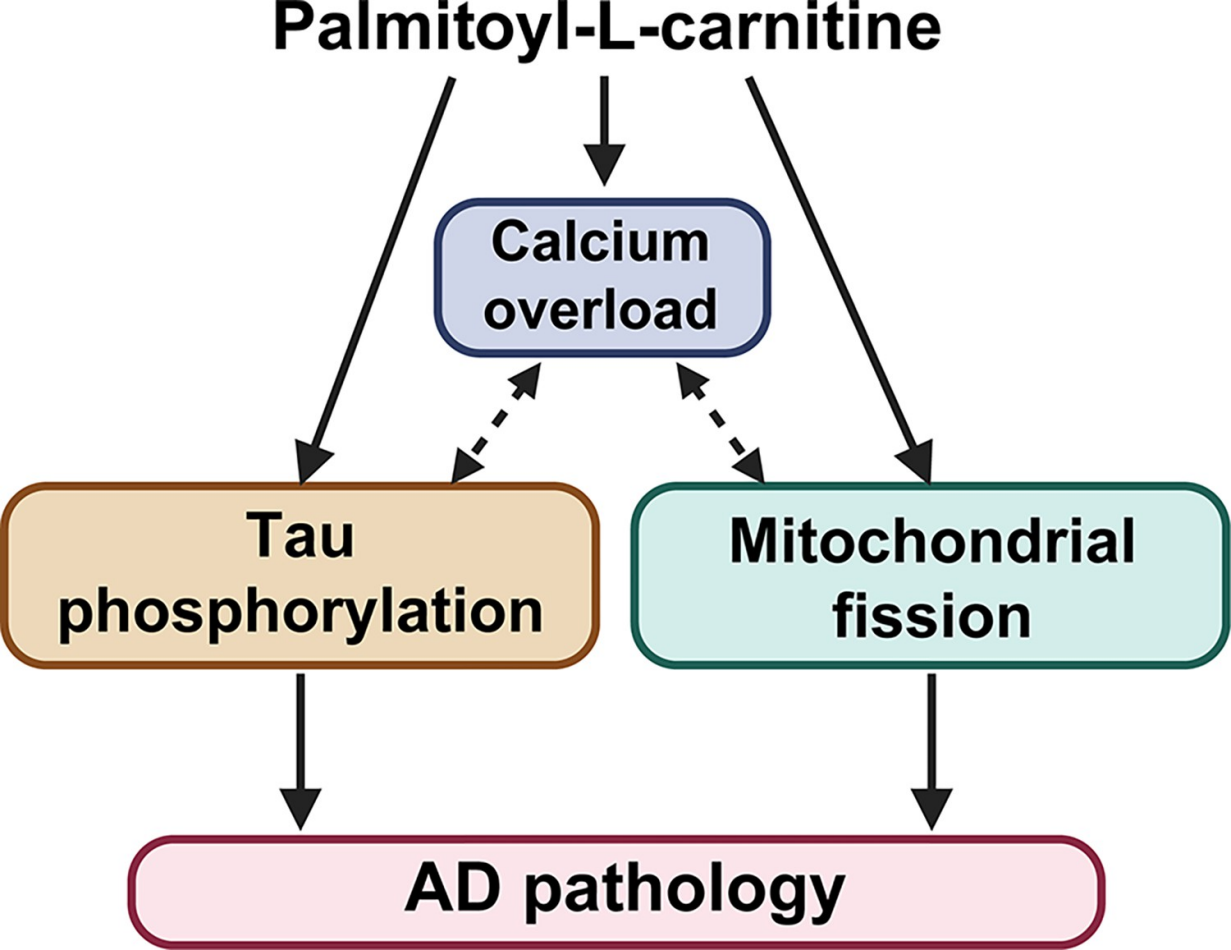
Palmitoyl-L-carnitine might play a key role in AD pathology with aging. A schematic illustration showing the mechanism by which palmitoyl-L-carnitine induces tau phosphorylation in SH-SY5Y neurons. Palmitoyl-L-carnitine causes calcium overload by closely interacting with mitochondrial malfunction, including the fission process. This increased calcium overload activates tau kinases (GSK-3β and CDK5/p25), leading to significant tau phosphorylation. Therefore, elevated serum levels of palmitoyl-L-carnitine are likely to contribute significantly to the development of AD pathology with aging.

Our findings elucidate an intricate relationship between abnormal serum lipids, particularly palmitoyl-L-carnitine, and neurodegenerative processes, highlighting a potential mechanism through which lipid dysregulation exacerbates conditions like AD. Specifically, we observed an age-related increase in serum palmitoyl-L-carnitine, which affects mitochondrial fission, calcium overload, and tau phosphorylation—key factors in mitochondrial dysfunction and tau pathology (Fig 6). This aligns with previous studies demonstrating that mitochondrial dysfunction exacerbates tau pathology by promoting tau hyperphosphorylation and aggregation, which lead to the neurofibrillary tangles characteristic of AD [39]. Notably, although alterations in levels of various acylcarnitine, including both decreases and increases, have been reported in AD patients [40, 41], the role of palmitoyl-L-carnitine had not been explored before.

Previous research identified the reduction of carnitine palmitoyl-transferase—an enzyme crucial for the beta-oxidation of long-chain acylcarnitines—in AD pathology [42], suggesting the potential accumulation of palmitoyl-L-carnitine. Our study is the first to directly investigate and confirm this accumulation with aging. Furthermore, we show that palmitoyl-L-carnitine contributes to tau phosphorylation along with mitochondrial dysfunction, establishing a direct link between altered acylcarnitine metabolism and a mechanistic pathway involved in AD pathology. This highlights the novelty of our research in addressing a previously unexamined aspect of lipid dysregulation in AD.

Although the exact sequence of events in the mechanism of palmitoyl-L-carnitine is not fully validated, its role in tau phosphorylation likely stems from mitochondrial dysfunction. Altered acylcarnitines, such as palmitoyl-L-carnitine, disrupt energy metabolism and beta-oxidation, leading to mitochondrial fragmentation and increased cytosolic calcium [43–45]. This increase in calcium activates calpain, inducing a conformational change that shifts it into an active state and promotes CDK5 activation through the cleavage of p35 into p25 [46, 47]. Both calpain and CDK5 contribute to GSK-3β activation: calpain by cleaving its inhibitors and CDK5 through direct phosphorylation, which phosphorylates DRP1, promoting mitochondrial fission [37, 48, 49]. Given that CDK5 and GSK-3β are strong tau kinases [50], this vicious cycle induced by palmitoyl-L-carnitine may result in a feedback loop of mitochondrial dysfunction and tau hyperphosphorylation, exacerbating AD pathology.

Although our study focused on neurons, palmitoyl-L-carnitine may affect tau pathology in the brain through alternative pathways like inflammation-mediated kinase activation. Mitochondrial dysfunction recruits IKK complex, leading to NF-κB-mediated transcription of pro-inflammatory cytokines like TNF-α and IL-6, which are associated with GSK-3β activity [51, 52]. Furthermore, mitochondrial dysfunction promotes NLRP3 inflammasome assembly and caspase-1 activation, leading to the secretion of pro-inflammatory cytokines like IL-1β, which are associated with the aberrant activation of CDK5 [53, 54]. Thus, to our knowledge, palmitoyl-L-carnitine may worsen tau pathology through direct metabolic effects and inflammation-mediated kinase activation, highlighting its multifaceted role in neuroinflammation, oxidative stress, and kinase dysregulation associated with AD. Further research is needed to fully elucidate these pathways and their broader implications for AD pathogenesis.

Existing studies have reported significant alterations in specific lipid metabolites associated with AD, although the exact species and their roles in the pathogenesis remain controversial. For instance, decreased levels of phosphatidylcholines, which are crucial for maintaining cell membrane integrity and synaptic function, have been observed in AD patients [13]. Additionally, elevated levels of sphingomyelins, which promote neuroinflammation and apoptosis, have also been reported in AD patients [55]. In our study, we observed dysregulation of serum phosphatidylcholines and sphingomyelins with aging in mice (Fig 1). These findings suggest a potential link between the altered lipid metabolome in aging and the lipid dysregulation observed in AD, highlighting the need to further investigate how age-related lipid changes may contribute to AD pathology.

Our findings of increased serum palmitoyl-L-carnitine with aging are consistent with observations in heart failure and coronary artery disease [56], where elevated levels likely result from disrupted mitochondrial fatty acid oxidation and impaired β-oxidation, leading to acylcarnitine accumulation [57]. Additionally, tissue damage and cell death associated with cardiovascular diseases can release intracellular contents into the bloodstream [58], further increasing serum palmitoyl-L-carnitine levels. This accumulation reflects not only metabolic dysfunction and oxidative stress but also suggests a broader systemic disruption that may contribute to the pathogenesis of various age-related disorders, including central nervous system diseases [59]. Therefore, investigating whether palmitoyl-L-carnitine accumulation driven by cardiovascular and other metabolic diseases, plays a fundamental role in age-related conditions could provide insights into its potential as a biomarker for underlying systemic metabolic disruptions. Further research is needed to explore the broader implications of palmitoyl-L-carnitine in various age-related pathologies.

MFF recruits both phosphorylated and non-phosphorylated forms of DRP1 to the mitochondrial membrane, facilitating fission independently of DRP1’s phosphorylation status [60]. Our study showed that palmitoyl-L-carnitine influences mitochondrial fission by promoting the phosphorylation of DRP1 and MFF, increasing MFF expression, without altering DRP1 protein levels (Fig 3). This aligns with evidence that mitochondrial fission can proceed without changes in DRP1 expression, such as with deletion of PTEN-induced kinase 1 or protein kinase A activation, which affect DRP1 phosphorylation at Ser616 without altering protein levels [61, 62]. Similarly, CDK1 phosphorylates DRP1 at Ser616 during mitosis without altering DRP1 levels [63]. Given palmitoyl-L-carnitine’s broad role in cellular metabolism and mitochondrial function, its induction of mitochondrial fission may involve kinases such as PTEN-induced kinase 1, protein kinase A, and CDK1 [64–66]. Further investigations are needed to elucidate how DRP1 expression remains unchanged despite these modifications.

Mitochondrial fission and calcium overload are closely interconnected, influencing each other and significantly impacting cellular homeostasis and pathology. Elevated intracellular calcium activates enzymes such as calcineurin, a calcium-activated phosphatase, that promotes mitochondrial fission by dephosphorylating and activating DRP1 at the mitochondrial membrane [67–70]. Conversely, excessive mitochondrial fission can disrupt calcium homeostasis, leading to calcium release into the cytosol and exacerbating cellular stress [71]. This interplay contributes to neuronal dysfunction and apoptosis in neurodegenerative diseases like AD, and cardiomyocyte apoptosis in cardiovascular diseases [72, 73]. Therefore, factors like palmitoyl-L-carnitine, which affect both mitochondrial dysfunction and calcium imbalance, may accelerate disease progression.

SH-SY5Y cells are commonly used as an in vitro model for studying neurodegenerative diseases like AD [74]. However, they have certain limitations. Derived from human neuroblastoma, these cells do not fully replicate the complex environment of human brain neurons, as they lack cellular interactions, three-dimensional structure, and supporting glial cells [75–77]. Their differentiation status and phenotypic stability can vary with culture conditions, which may potentially affect results [78]. Consequently, findings from SH-SY5Y cells may not fully translate to in vivo conditions or capture the complex pathophysiology of the human brain. Thus, while our in vitro results provide valuable insights, further validation in in vivo models or human-derived neurons is necessary to confirm their clinical relevance.

In conclusion, our study identifies a significant link between age-related increases in serum palmitoyl-L-carnitine levels, tau hyperphosphorylation, and mitochondrial fission, revealing a novel pathway through which lipid metabolism dysregulation affects neurodegenerative processes. We found that mitochondrial dysfunction induced by palmitoyl-L-carnitine leads to calcium overload and activation of kinases that contribute to tau hyperphosphorylation. This underscores the therapeutic potential of targeting lipid metabolism, including reducing serum palmitoyl-L-carnitine levels, in AD and other neurodegenerative diseases. Our findings highlight the need to develop supplements or interventions to lower palmitoyl-L-carnitine synthesis or levels as a promising treatment approach. Further research should explore the mechanisms by which palmitoyl-L-carnitine and related acylcarnitines affect mitochondrial dynamics, kinase activity, inflammation, and other pathways involved in tau pathology. Ultimately, the result suggests that palmitoyl-L-carnitine could be a novel therapeutic target for treating neurodegenerative diseases including AD.

## Supporting information

S1 File Uncropped full blots corresponding to cropped bands present in the result section. (A) is the uncropped full blot corresponding to the cropped bands in Fig 2. (B) is the uncropped full blot corresponding to the cropped bands in Fig 3. (C) is the uncropped full blot corresponding to the cropped bands in Fig 5A. (D) is the uncropped full blot corresponding to the cropped bands in Fig 5B. In (A) to (D), capital letter X indicates samples that were not used as representative images in each respective figure. The numbers on the left side of the uncropped full blots represent the molecular weight ladder. The right tortoise shell bracket on the uncropped full blots marks the cropped region.
(PDF)
