# Supplementary material for: Palmitoyl-L-carnitine induces tau phosphorylation and mitochondrial dysfunction in neuronal cells
Source: PLoS One. 2024 Nov 13;19(11):e0313507. doi: 10.1371/journal.pone.0313507 (PMC11560007; doi:10.1371/journal.pone.0313507)

Supplementary information

Palmitoyl-L-carnitine induces tau phosphorylation and mitochondrial dysfunction in neuronal cells

Gwangho Yoon, Min Kyoung Kam, Young Ho Koh, and Chulman Jo*

This S1 File contains uncropped full blots

A. Full scans of uncropped blots (For Fig 2)

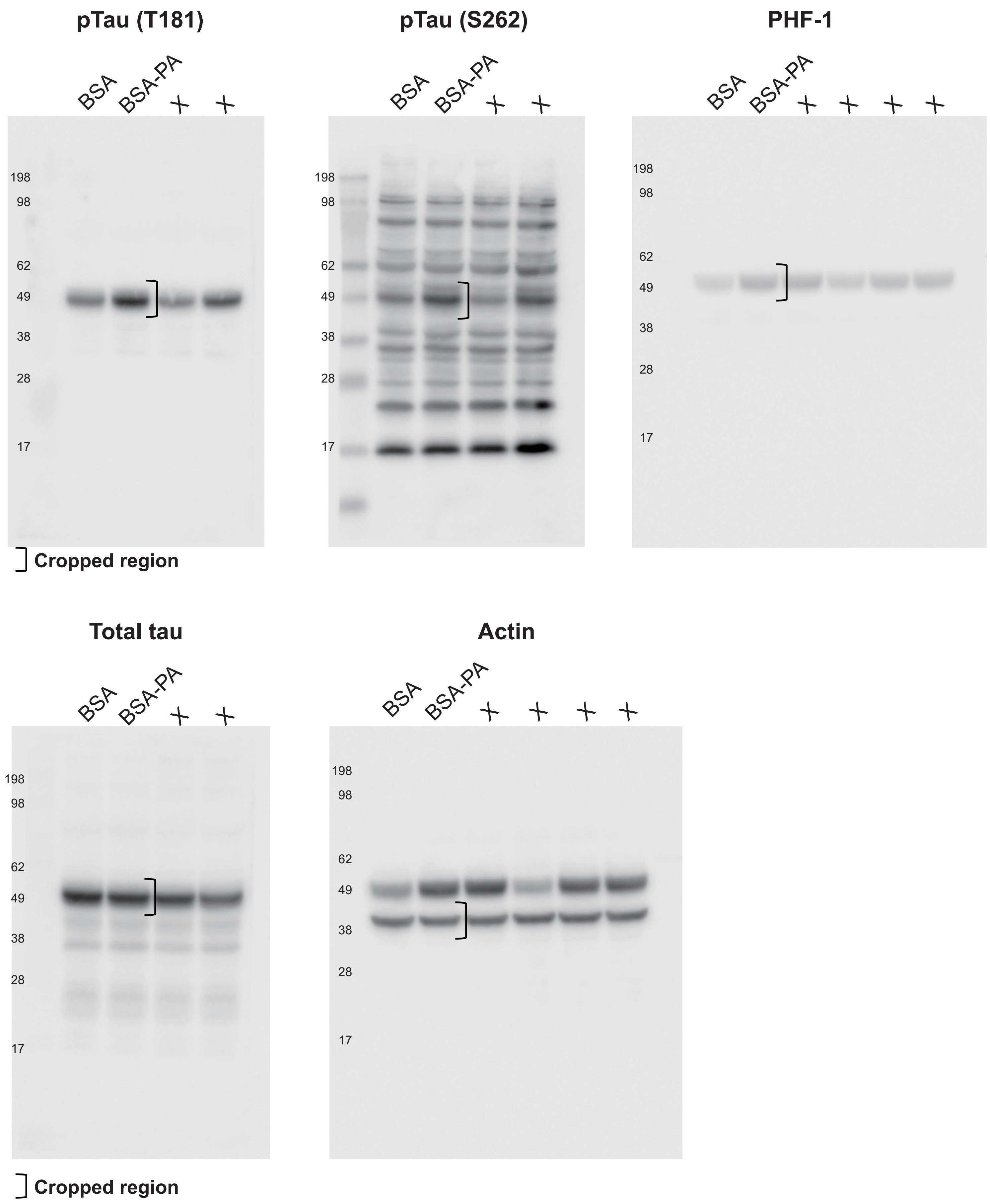

B. Full scans of uncropped blots (For Fig 3)

For Fig3A

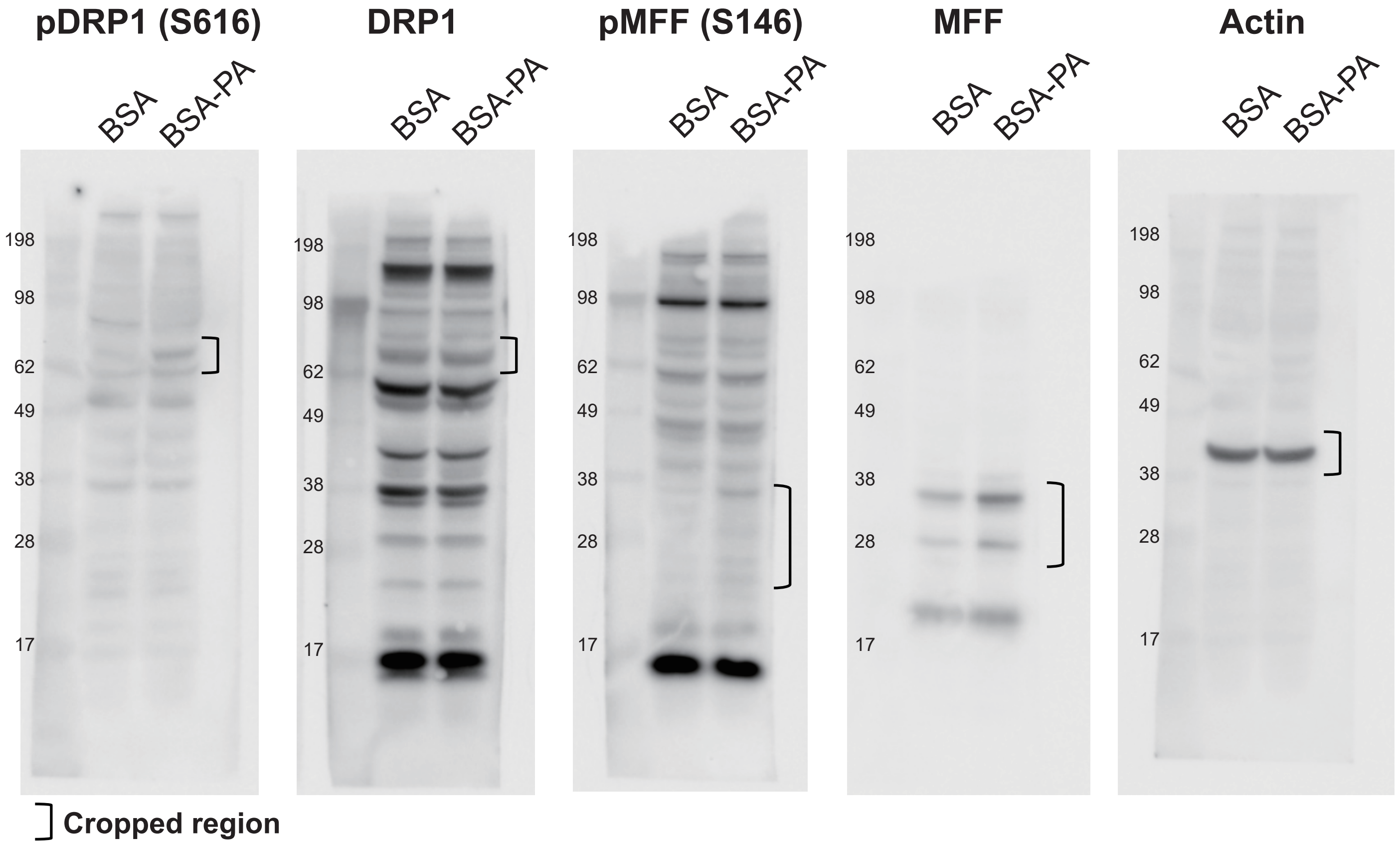

For Fig3C

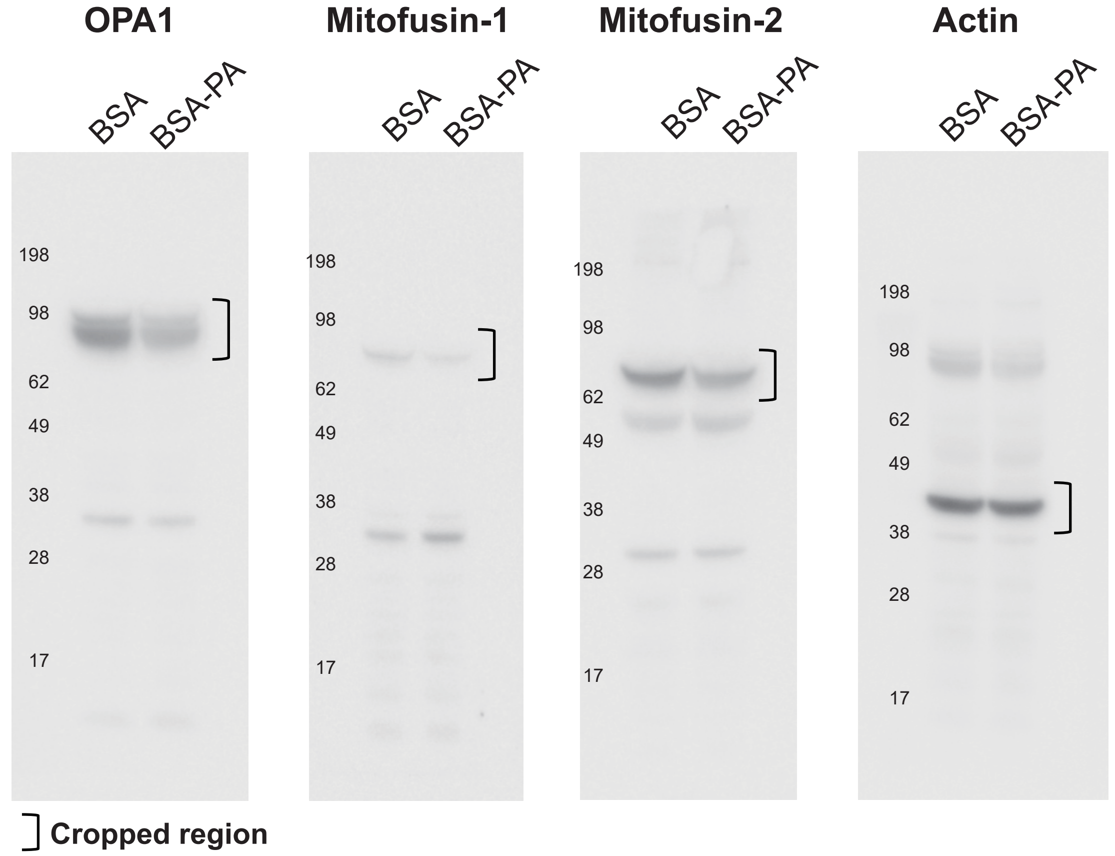

C. Full scans of uncropped blots (For Fig 5A)

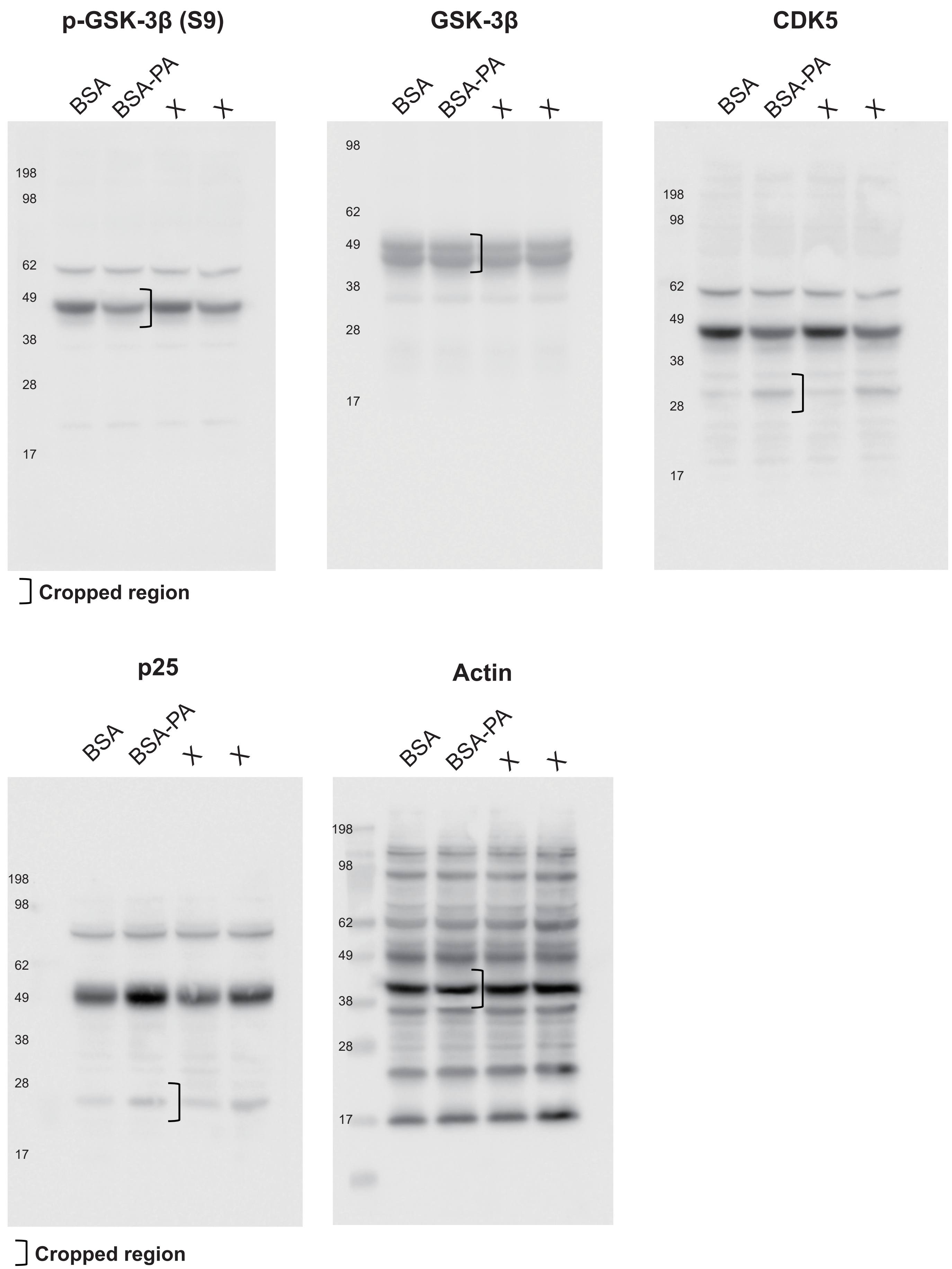

D. Full scans of uncropped blots (For Fig 5B)

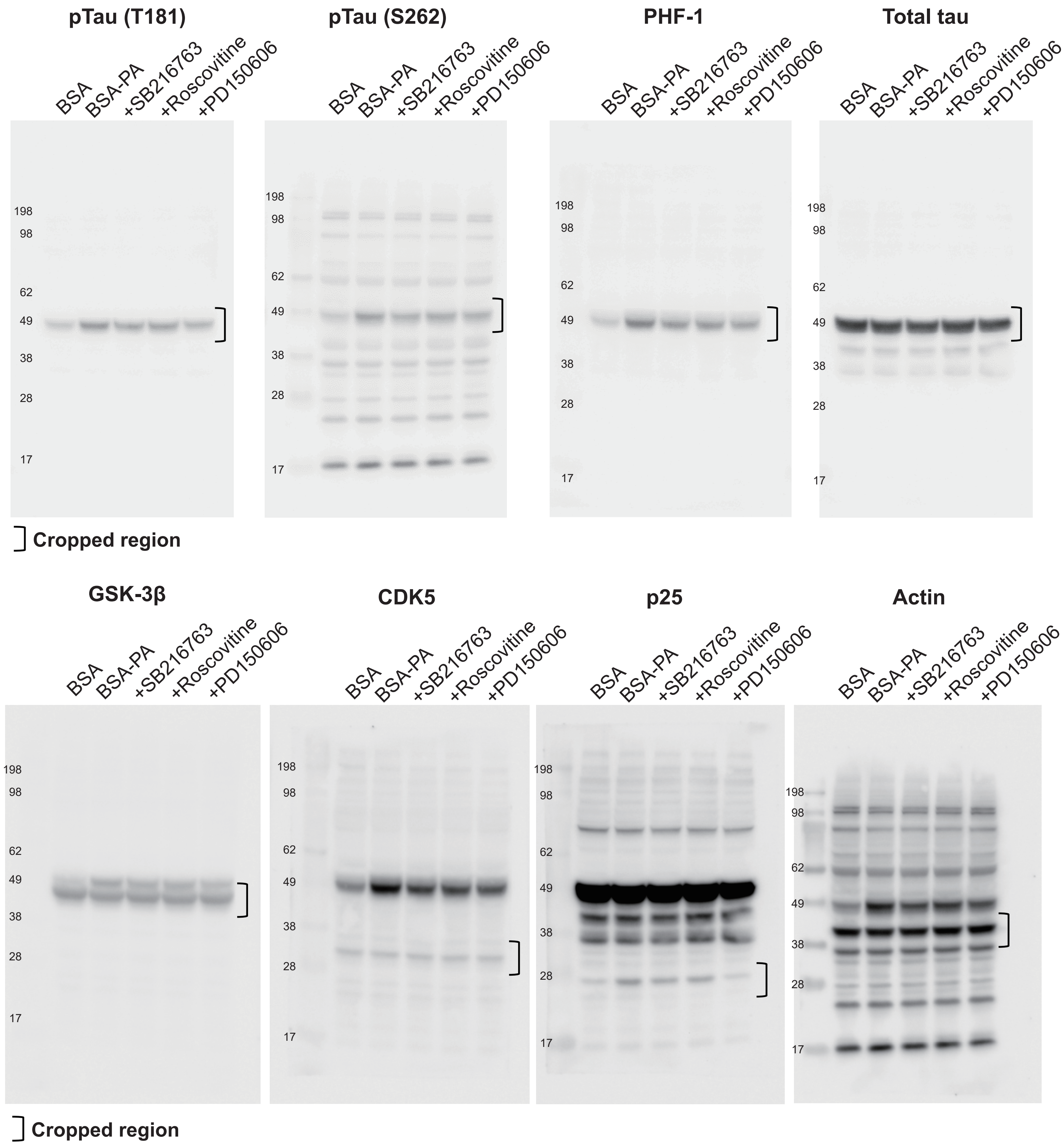

Supplement: S1 File — (A) is the uncropped full blot corresponding to the cropped bands in Fig 2. (B) is the uncropped full blot corresponding to the cropped bands in Fig 3. (C) is the uncropped full blot corresponding to the cropped bands in Fig 5A. (D) is the uncropped full blot corresponding to the cropped bands in Fig 5B. In (A) to (D), capital letter X indicates samples that were not used as representative images in each respective figure. The numbers on the left side of the uncropped full blots represent the molecular weight ladder. The right tortoise shell bracket on the uncropped full blots marks the cropped region. (PDF) [file pone.0313507.s001.pdf]
